# Elevated body roundness index and epilepsy prevalence: a cross-sectional study

**DOI:** 10.1038/s41598-026-36062-8

**Published:** 2026-01-19

**Authors:** Tieshi Zhu, Zhiwei Long, Saihui Zhu, Hui Mai

**Affiliations:** 1 Department of Neurology, Zhanjiang Central Hospital, Guangdong Medical University, Zhanjiang, Guangdong China; 2 Guangzhou Medical University, Guangzhou, Guangdong China; 3 School of Pharmacy, China Medical University, Shenyang, Liaoning China

**Keywords:** BRI, NHANES, Central obesity, Epilepsy, Cross-sectional study, Neurology, Risk factors

## Abstract

Obesity and epilepsy have a complex bidirectional relationship. Body mass index is commonly used to study this association but does not capture fat distribution. The Body Roundness Index (BRI) reflects abdominal adiposity. To our knowledge, the association between BRI and epilepsy prevalence has not been reported. We analyzed 17,914 adults from NHANES 2013–March 2020. BRI was modeled continuously and by tertiles. We used logistic regression to estimate odds ratios (ORs) and 95% CIs for prevalent epilepsy. Restricted cubic spline analyses evaluated nonlinearity. Prespecified sensitivity analyses excluded participants taking valproate (n = 6) or carbamazepine (n = 23). Variance inflation factors assessed multicollinearity. No nonlinear relationship between BRI and epilepsy (P = 0.609). Higher BRI was associated with greater odds of prevalent epilepsy in fully adjusted models (per-unit OR, 1.08; 95% CI 1.01–1.15; P = 0.03). Compared with Q1, Q3 showed higher odds (OR, 1.73; 95% CI 1.06–2.89; P = 0.03), whereas Q2 did not reach significance (OR, 1.58; 95% CI 0.97–2.63; P = 0.07). Findings were materially unchanged after excluding valproate or carbamazepine users. Subgroup point estimates exceeded 1 across strata; tests for interaction were not significant. Higher BRI is associated with higher prevalence of epilepsy. These results underscore the potential importance of abdominal fat distribution in epilepsy and warrant confirmation in larger prospective studies.

**Supplementary Information:**

The online version contains supplementary material available at 10.1038/s41598-026-36062-8.

## Introduction

Epilepsy is a prevalent and severe chronic neurological disorder globally, characterized by abnormal brain activity that leads to seizures, along with behavioral and sensory disturbances, and, in some cases, loss of consciousness^1–3^. Patients with epilepsy have a higher likelihood of obesity, and conversely, several studies have also reported that individuals with obesity have an increased risk of epilepsy^4–7^. Furthermore, maternal obesity has been linked to a higher risk of epilepsy in infants^8^. These findings highlight the complex interplay between epilepsy and obesity.

For many years, body mass index (BMI) has been commonly used to assess obesity severity, with a BMI ≥ 30 (kg/m^2^) typically classified as obese and a BMI between 25 and 30 as overweight^9,10^. Studies have shown that seizure frequency increases significantly in individuals with a BMI < 18.5 or BMI ≥ 40^11^, and a BMI ≥ 25 is associated with a higher risk of drug-resistant epilepsy (DRE) in adults^12^. These findings suggest that BMI is a useful tool for evaluating the relationship between obesity and epilepsy. However, it is important to note that BMI is a crude measure that only reflects the ratio of weight to height; it cannot differentiate between muscle and fat mass, nor does it provide insight into the distribution of body fat. In 2013, U.S. researchers introduced the Body Roundness Index (BRI), which is based on height and waist circumference. Compared to BMI, BRI more accurately reflects abdominal fat distribution, and its relationship with cardiovascular diseases has been widely studied^13–15^. However, studies examining the association between BRI and epilepsy risk remain scarce. Therefore, this study aims to analyze the relationship between BRI and epilepsy risk using data from the National Health and Nutrition Examination Survey (NHANES).

## Results

### The baseline characteristics of participants

Table 1 presents the baseline characteristics of participants across the BRI tertiles (Q1–Q3). The number of participants in each group was similar, but significant differences were observed in baseline characteristics across groups (p < 0.05). Mean BRI values were 3.22, 5.22, and 8.35, respectively. Participants in the Q3 and Q2 groups tended to have a higher average age. The Q3 group had a greater proportion of females and white individuals, as well as the lowest mean the ratio of family income to poverty (PIR) and education levels. From Q1 to Q3, the prevalence of diabetes and hypertension increased, with Q3 showing the highest prevalence of both conditions. The Q3 group also had the lowest proportion of non-smokers but a smaller proportion of current drinkers compared to the other groups. Finally, the proportion of participants with epilepsy was higher in Q2 and Q3 than in Q1, with the highest proportion observed in the Q3 group (Table 1).

**Table 1:** Baseline characteristics of participants according to BRI tertiles.

	Q1 (n = 5970) [1.17, 4.29]	Q2 (n = 5971) (4.29, 6.22]	Q3 (n = 5973) (6.22, 23.48]	*P*
Age (year)	40.27 (17.06)	51.20 (17.14)	51.95 (16.94)	< 0.001
Female	2754 (46.1%)	2700 (45.2%)	3624 (60.7%)	< 0.001
Race				< 0.001
White	2193 (36.7%)	2245 (37.6%)	2383 (39.9%)	
Black	1413 (23.7%)	1120 (18.8%)	1442 (24.1%)	
other	2364 (39.6%)	2606 (43.6%)	2148 (36.0%)	
PIR	2.66 (1.69)	2.69 (1.64)	2.38 (1.56)	< 0.001
Education				< 0.001
< College	4063 (68.1%)	4401 (73.7%)	4895 (82.0%)	
College or more	1907 (31.9%)	1570 (26.3%)	1078 (18.0%)	
BRI	3.22 (0.71)	5.22 (0.55)	8.35 (2.05)	< 0.001
Diabetes	331 (5.5%)	1031 (17.3%)	1882 (31.5%)	< 0.001
Hypertension	1328 (22.2%)	2653 (44.4%)	3410 (57.1%)	< 0.001
Smoke				< 0.001
never	3743 (62.7%)	3402 (57.0%)	3333 (55.8%)	
former	918 (15.4%)	1466 (24.6%)	1640 (27.5%)	
current	1309 (21.9%)	1103 (18.5%)	1000 (16.7%)	
Drink				< 0.001
never	839 (14.1%)	855 (14.3%)	852 (14.3%)	
former	315 (5.3%)	516 (8.6%)	615 (10.3%)	
current	4816 (80.7%)	4600 (77.0%)	4506 (75.4%)	
Epilepsy	27 (0.5%)	45 (0.8%)	56 (0.9%)	0.006

Values are presented as mean (SD) for continuous variables and n (%) for categorical variables. BRI tertiles were defined as Q1 [1.17–4.29], Q2 (4.29–6.22], and Q3 (6.22–23.48]. Abbreviations: BRI, body roundness index; PIR, the ratio of family income to poverty.

### Association of BRI with epilepsy prevalence

**Fig. 1:**
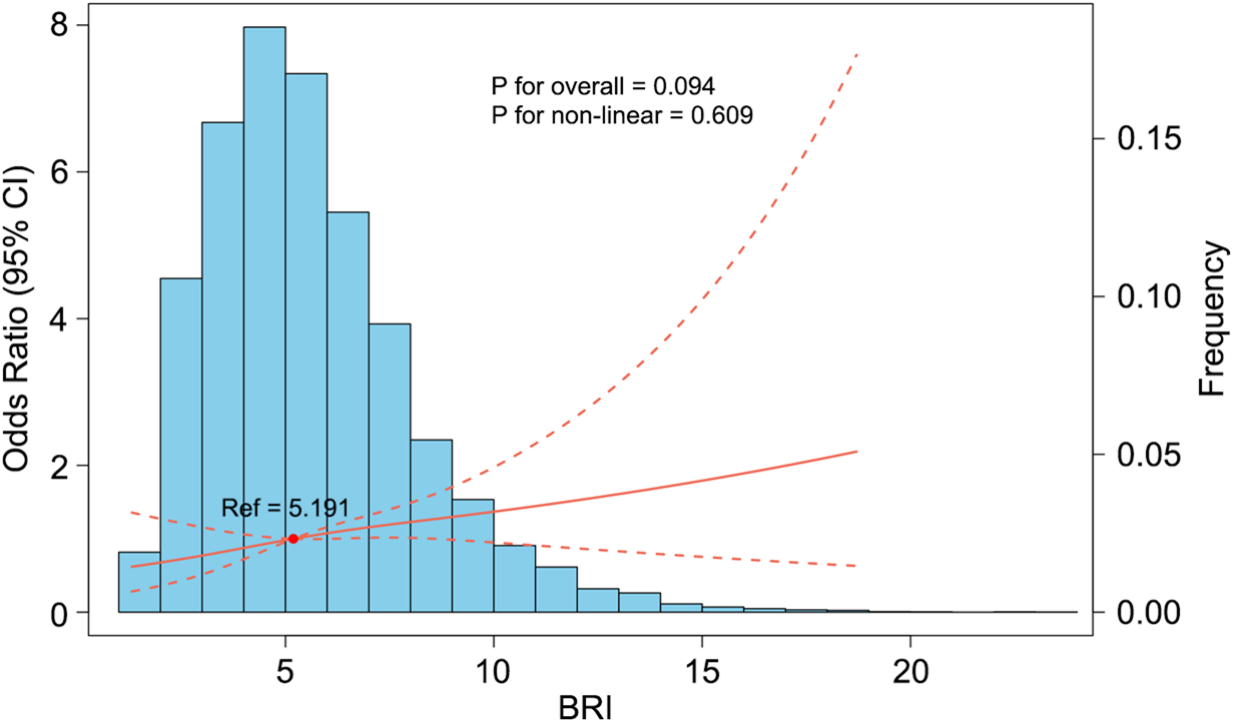
Restricted cubic spline showing the association between BRI and the odds of epilepsy. The solid red line represents the adjusted odds ratio and the dashed lines indicate 95% confidence intervals, with BRI = 5.191 as the reference. The blue bars depict the distribution of BRI in the study population. P values for the overall association and for nonlinearity are shown in the plot. Abbreviations: BRI, body roundness index.

The restricted cubic spline (RCS) analysis revealed no evidence of a nonlinear relationship between BRI and epilepsy (P for nonlinearity = 0.609, P for overall association = 0.094). The RCS plot closely resembled a straight line, indicating that epilepsy prevalence increased steadily with higher BRI. Additionally, most participants had a BRI within the range of 2–8, while relatively fewer individuals had a BRI exceeding 10 (Fig. 1).

**Fig. 2:**
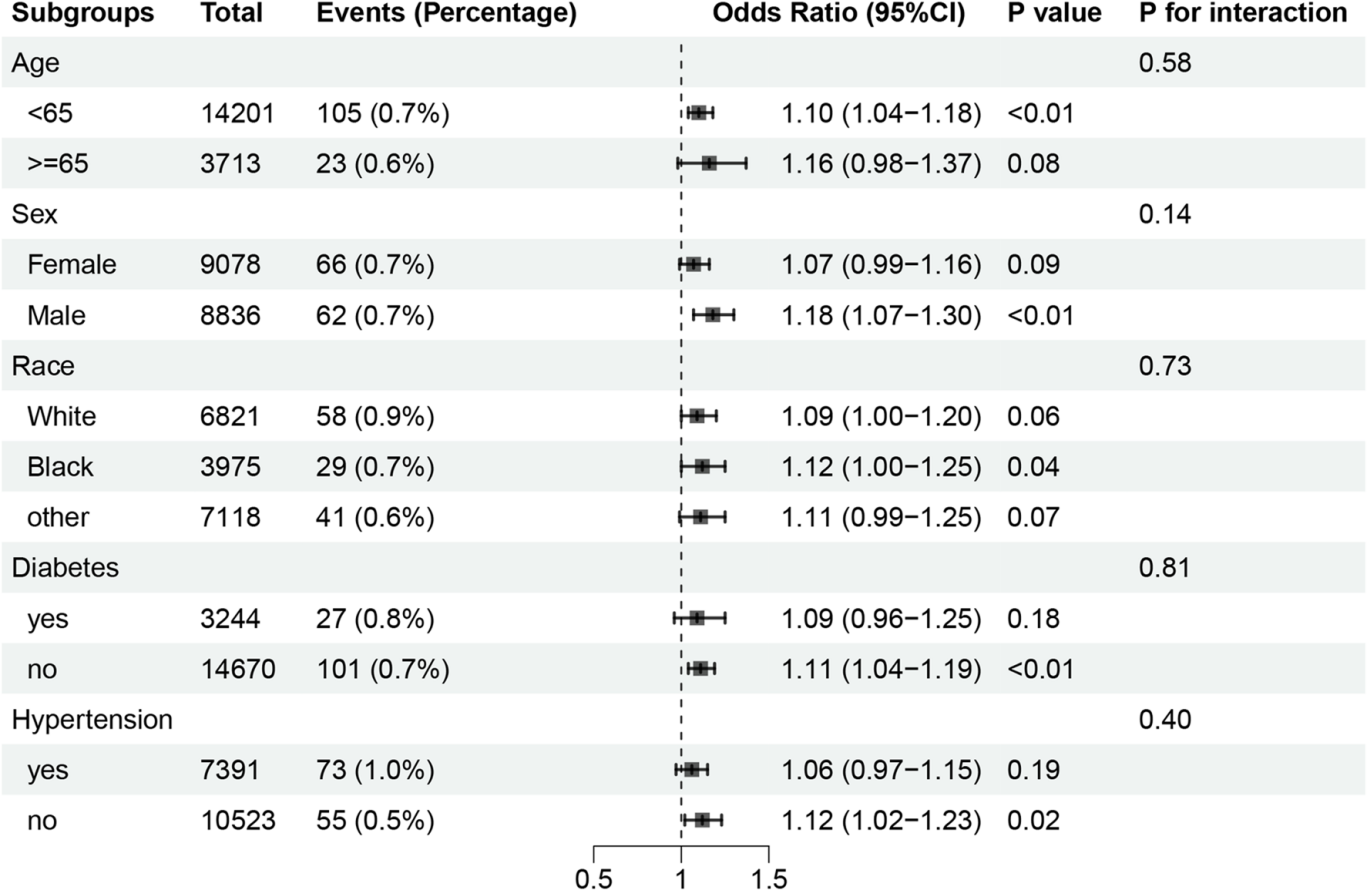
Subgroup analyses of the association between BRI and epilepsy. Points and horizontal lines represent adjusted odds ratios and 95% confidence intervals for epilepsy per 1-unit increase in BRI within each subgroup, with models adjusted as in Model 4 except for the stratifying variable. Columns show the total number of participants, number of epilepsy events (percentage), odds ratio (95% CI), P value for the association, and P for interaction. Abbreviations: BRI, body roundness index; CI, confidence interval.

Logistic regression analysis demonstrated that higher BRI was significantly associated with greater odds of prevalent epilepsy, with odds ratios (OR) above 1 and P-values below 0.05 across Models 1–4. In Model 4, which adjusted for all covariates, the association remained significant [OR 1.08, 95% confidence intervals (CI) 1.01–1.15, P = 0.03] (Table 2). Variance inflation factors (VIF) for all covariates were < 5, indicating no concerning multicollinearity (Table S1). Sensitivity analyses excluding participants reporting valproate use (n = 6) or carbamazepine use (n = 23) yielded materially unchanged estimates (Tables S2 and S3). Subgroup analyses showed that although the OR exceeded 1 in all subgroups, statistical significance (P < 0.05) was observed only in subgroups such as age < 65, male, Black, no diabetes, and no hypertension. In contrast, subgroups such as age ≥ 65, female, White, other races, diabetes, and hypertension did not show significant associations (Fig. 2). Tests for interaction by age, sex, race, diabetes, and hypertension were not statistically significant.

**Table 2:** Association between BRI and epilepsy in logistic regression models.

	OR	95%CI	P
Model1	1.11	(1.04,1.17)	< 0.001
Model2	1.11	(1.04,1.17)	0.001
Model3	1.09	(1.02,1.16)	0.01
Model4	1.08	(1.01,1.15)	0.03

ORs and 95% CIs for epilepsy are shown per 1-unit increase in BRI. Model 1 is unadjusted; Model 2 is adjusted for age, sex, and race; Model 3 is additionally adjusted for education level, the ratio of family income to poverty, smoking status, and alcohol consumption; Model 4 is further adjusted for diabetes and hypertension. Abbreviations: BRI, body roundness index; OR, odds ratio; CI, confidence interval.

When BRI was categorized into tertiles and reanalyzed, results were similar but slightly attenuated. Compared with Q1, both Q2 and Q3 showed higher odds of prevalent epilepsy in Models 1–3. However, in Model 4, only Q3 was significantly associated with an increased epilepsy risk (OR 1.73, 95% CI 1.06–2.89, P = 0.03), while Q2 did not reach statistical significance (OR 1.58, 95% CI 0.97–2.63, P = 0.07) (Table 3). Sensitivity analyses excluding participants reporting valproate use (n = 6) or carbamazepine use (n = 23) yielded materially unchanged tertile-based estimates (Table S4 and S5).

**Table 3:** Association between BRI tertiles and epilepsy in logistic regression models.

	BRI	OR	95%CI	*P*
Model 1	Q1	Ref	Ref	Ref
	Q2	1.67	(1.04,2.73)	0.04
	Q3	2.08	(1.33,3.35)	0.003
Model 2	Q1	Ref	Ref	Ref
	Q2	1.69	(1.04,2.79)	0.04
	Q3	2.07	(1.29,3.38)	0.003
Model 3	Q1	Ref	Ref	Ref
	Q2	1.68	(1.03,2.78)	0.04
	Q3	1.92	(1.19,3.17)	0.01
Model 4	Q1	Ref	Ref	Ref
	Q2	1.58	(0.97,2.63)	0.07
	Q3	1.73	(1.06,2.89)	0.03

ORs and 95% CIs for epilepsy are shown for BRI tertiles, with Q1 [1.17–4.29] as the reference group, Q2 (4.29–6.22], and Q3 (6.22–23.48]. Model 1 is unadjusted; Model 2 is adjusted for age, sex, and race; Model 3 is additionally adjusted for education level, the ratio of family income to poverty, smoking status, and alcohol consumption; Model 4 is further adjusted for diabetes and hypertension. Abbreviations: BRI, body roundness index; OR, odds ratio; CI, confidence interval.

## Discussion

In this study, using data from NHANES 2013–2020, we report for the first time that the BRI is positively associated with the prevalence of epilepsy, with higher BRI corresponding to a higher prevalence of epilepsy. This association remained robust in sensitivity analyses, and no significant interactions were observed across subgroups; although estimates reached significance in men and Black participants but not in other strata, these differences likely reflect limited power and residual sociodemographic confounding. Consistent with this, all P for interaction were > 0.05, indicating no evident effect modification. Because BRI incorporates waist circumference and better captures central obesity than BMI, its correlation with epilepsy underscores the need to pay greater attention to abdominal fat when considering epilepsy risk and management.

Numerous studies have explored the relationship between obesity and epilepsy, revealing a complex and bidirectional association. First, obesity rates are higher among individuals with epilepsy compared to those without. A study of 26,266 adolescents (10–17 years) reported higher obesity odds in participants with epilepsy/seizure disorders (OR, 2.2; 95% CI 1.2–3.8)^16^. Similarly, in adults, a cross-sectional study including 14,246,785 respondents aged 19–110 years reported that epilepsy was significantly associated with an increased incidence of obesity^17^. This trend is also observed in children. A study of 446 children with childhood absence epilepsy (CAE) and 2,079 age- and sex-matched controls noted a higher prevalence of overweight and obesity among children with newly diagnosed CAE^18^. Moreover, certain antiepileptic drugs, such as valproate (VPA), have been shown to increase the likelihood of obesity^19^. Second, obesity may increase the risk of epilepsy, though this remains controversial^20,21^. A retrospective cohort study reported a significantly higher rate of seizures in individuals with a BMI ≥ 40^11^. Another study demonstrated that a BMI ≥ 25 increased the risk of DRE in adults^12^. Additionally, maternal obesity has been associated with a higher risk of epilepsy in offspring^8^. To investigate the causal relationship between obesity and epilepsy, some researchers have employed Mendelian randomization, though findings are inconsistent. A two-sample Mendelian randomization study suggested that obesity is a risk factor for epilepsy, and increased waist circumference is specifically linked to a higher risk of adolescent myoclonic epilepsy^7^. In contrast, a study by Wang et al. reported no causal relationship between BMI and epilepsy^22^. In this study, we found that an increased BRI was associated with a higher odds of prevalent epilepsy. Since BRI is calculated using height and waist circumference, our findings align more closely with those of Zhou et al.^7^. However, this study does not establish a causal relationship between BRI and epilepsy, highlighting only a correlation.

The mechanisms by which obesity may increase epilepsy risk remain incompletely understood. Structural studies indicate that greater adiposity is associated with hippocampal volume loss, with more pronounced atrophy at higher BMI and a predilection for the left hippocampus^23^. Beyond gross morphology, obesity can mediate neuroinflammation and mitochondrial dysfunction, affecting the structure and function of the cortex, brainstem, and amygdala^24,25^. Beyond morphology, obesity-related metabolic and vascular disturbances—including insulin resistance, hyperglycemia, endothelial dysfunction, and mitochondrial impairment—can disrupt hippocampal and network excitability^26^. In addition, abdominal adiposity is closely linked to systemic inflammation, with elevations in proinflammatory cytokines (eg, IL-6, TNF-α) that have been associated with seizure occurrence and recurrence^27–29^. Given the well-established link between neuroinflammation and epilepsy, with inflammatory factors playing a significant role in epileptogenesis, inflammation has been proposed as a potential therapeutic target for epilepsy^30–32^. Obesity is also associated with increased cardiovascular disease burden, and these comorbidities are themselves risk factors for epilepsy^33–35^. Taken together, these observations support the hypothesis that obesity may contribute to epileptogenesis through convergent neuroinflammatory, metabolic, and vascular pathways.

BMI has been widely used as a primary indicator of obesity in many of the studies mentioned earlier. However, BMI is a simplistic measure based solely on height and weight, which fails to distinguish between muscle and fat or accurately assess fat distribution, particularly visceral fat accumulation. This limitation is especially relevant for individuals with central obesity, often colloquially described as “middle-aged greasiness”. To address these shortcomings, the BRI was developed. BRI quantifies the roundness of an individual’s body by incorporating waist circumference and height, with a particular emphasis on central obesity. This index provides a more precise assessment of health risks, especially for individuals who may not appear overweight but have significant visceral fat accumulation. The findings of this study highlight a positive relationship between visceral fat—indexed by BRI—and the prevalence of epilepsy. Unlike BMI, which has been reported to show a U-shaped association with epilepsy prevalence (eg, higher prevalence at BMI < 15 and ≥ 40)^11^, we observed that higher BRI is consistently associated with higher prevalence of epilepsy. These results suggest that BRI may be a more appropriate and accurate tool than BMI for evaluating epilepsy prevalence, particularly in the context of central (abdominal) obesity.

This study has several limitations. First, as a cross-sectional study, it can only demonstrate correlation and not causation. Second, BRI primarily focuses on abdominal fat, neglecting other forms of fat accumulation, such as in the buttocks and thighs. Third, the proportion of epilepsy cases in this sample was relatively low, which may lead to unstable estimates in subgroup analyses; larger, longitudinal cohort studies are needed to confirm these findings and clarify temporal relationships. Finally, the diagnosis of epilepsy in this study relied on self-reports from respondents, which may introduce reporting bias or inaccuracies.

Despite these limitations, this study is the first to identify a correlation between BRI and epilepsy, highlighting the importance of examining the relationship between abdominal fat and epilepsy. Future prospective studies are warranted to explore the impact of abdominal fat on seizure risk and frequency. Additionally, researchers should not solely rely on BMI as an indicator but should recognize its limitations and consider alternative measures, such as BRI, when analyzing the relationship between obesity and epilepsy.

## Methods

### Population

**Fig. 3:**
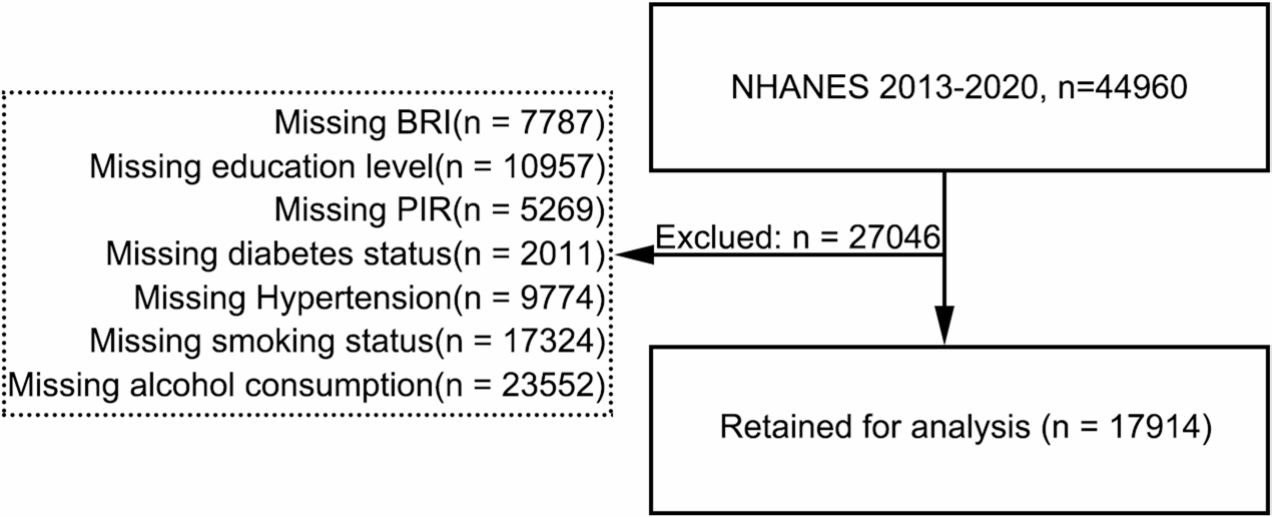
Flowchart of participant inclusion and exclusion process. Among 44,960 participants in NHANES 2013–2020, 27,046 were excluded due to missing information, leaving 17,914 participants for the final analysis. Abbreviations: NHANES, National Health and Nutrition Examination Survey; BRI, body roundness index; PIR, the ratio of family income to poverty.

This study analyzed data from 44,960 individuals extracted from NHANES 2013–2020. After excluding 27,046 participants with missing data (Fig. 3), a total of 17,914 individuals remained eligible for the final analysis. Among these, 128 participants were diagnosed with epilepsy.

### Outcome

Epilepsy-related information was collected through face-to-face interviews conducted by trained investigators. Participants were asked to provide details of all prescribed medications used in the past 30 days, along with the corresponding indications for each medication. The study classified participants based on whether they were taking one or more medications prescribed for “epilepsy and recurrent seizures” (ICD-10 code G40). Individuals who reported using such medications were classified as having epilepsy^36,37^.

### BRI

BRI was calculated using the formula developed by Thomas et al.^38,39^:

$$\begin{aligned} {\mathrm{BRI}} & = {364}.{2} - {365}.{5} \times \surd \left[ {{1} - \left( {{\mathrm{waist}}\;{\mathrm{in}}\;{\mathrm{centimeters}}/{2}\pi } \right)^{{2}} } \right. \\ & \quad \left. {/\left( {0.{5} \times {\mathrm{height}}\;{\mathrm{in}}\;{\mathrm{centimeters}}} \right)^{2} } \right] \\ \end{aligned}$$

In this study, BRI was categorized into three groups based on tertiles: Q1 [1.17, 4.29], Q2 (4.29, 6.22] and Q3 (6.22,23.48].

### Covariate

The covariates for this study included age, sex, race, PIR, education, diabetes, hypertension, smoking status, and alcohol consumption. Data on age, sex, race, PIR, and education were obtained directly from the official NHANES website.

Participants were classified as having diabetes if they met any of the following criteria: (1) a physician-diagnosed diabetes condition; (2) use of glucose-lowering medications or insulin therapy; (3) a random blood glucose level > 11.1 mmol/L; (4) a two-hour blood glucose level > 11.1 mmol/L during an oral glucose tolerance test; or (5) a hemoglobin A1c level > 6.5%^40,41^. Hypertension was defined as meeting any of the following criteria: (1) a physician diagnosis of hypertension; (2) use of antihypertensive medication; or (3) an average blood pressure ≥ 140/90 mmHg^42^. Smoking status was categorized as: “Never” (fewer than 100 cigarettes smoked in a lifetime), “Former” (smoked more than 100 cigarettes but had quit), and “Current” (smoked more than 100 cigarettes and were currently smoking)^43^. Alcohol consumption status was classified as: “Never” (consumed alcohol fewer than 12 times in a lifetime), “Former” (consumed alcohol more than 12 times in a lifetime but none in the past year), and “Current” (consumed alcohol more than 12 times in a lifetime and within the past year)^44,45^.

### Statistic

Data extraction and analysis were performed using R version 4.4.1. Continuous variables were analyzed using either Student’s t-test or the Mann–Whitney U test, depending on the distribution of the data, while categorical variables were assessed using the chi-square test or Fisher’s exact test. RCS analysis was employed to evaluate the nonlinear relationship between BRI and epilepsy. Knots were placed at the 5th, 35th, 65th, and 95th percentiles with the cohort median as the reference. Models adjusted for age, sex, race, education, PIR, smoking status, alcohol consumption, diabetes and hypertension; overall and nonlinearity were tested with Wald χ^2^ statistics.

The association between BRI and epilepsy was examined using logistic regression, with results presented as OR and 95% CI. Forest plots were constructed to visually represent these associations. Model 1 was unadjusted, Model 2 adjusted for age, sex, and race, Model 3 further adjusted for education, PIR, smoking status, and alcohol consumption, and Model 4 additionally adjusted for diabetes and hypertension. Multicollinearity was assessed using VIFs; values greater than 5 were considered indicative of multicollinearity.

In subgroup analyses, the same covariates as Model 4 were adjusted for, except for the variables defining the subgroups themselves. The covariates used in the RCS analysis were consistent with those in Model 4. A p-value of less than 0.05 was considered statistically significant.

### Sensitivity analysis

We assessed robustness by re-estimating the logistic models after excluding participants taking valproate (n = 6) and, separately, those taking carbamazepine (n = 23).

## Supplementary Information

Below is the link to the electronic supplementary material.

Supplementary Material 1

Supplementary Material 2

Supplementary Material 3

Supplementary Material 4

Supplementary Material 5

## Data Availability

The data for this study were obtained from the NHANES database, publicly accessible at https://www.cdc.gov/nchs/nhanes/.
