# Supplementary material for: Elevated body roundness index and epilepsy prevalence: a cross-sectional study
Source: Sci Rep. 2026 Jan 19;16:5685. doi: 10.1038/s41598-026-36062-8 (PMC12891725; doi:10.1038/s41598-026-36062-8)
Supplement: Supplementary file 1 — Supplementary Material 1 [file 41598_2026_36062_MOESM1_ESM.docx]

Table S1. VIFs for covariates in the fully adjusted logistic regression model

|  | VIF |
| --- | --- |
| BRI | 1.204 |
| Age | 1.506 |
| Sex | 1.102 |
| Race | 1.105 |
| PIR | 1.254 |
| Education | 1.187 |
| Smoke | 1.391 |
| Drink | 1.274 |
| Hypertension | 1.410 |
| Diabetes | 1.173 |

VIFs were calculated from the fully adjusted model (Model 4). Values <5 were considered not indicative of concerning multicollinearity. For categorical variables (e.g., race, education, smoking, alcohol use), indicators were included in the model and the highest VIF across levels is shown. Abbreviations: VIF, variance inflation factors; BRI, body roundness index; PIR, the ratio of family income to poverty.
