# Supplementary material for: Elevated body roundness index and epilepsy prevalence: a cross-sectional study
Source: Sci Rep. 2026 Jan 19;16:5685. doi: 10.1038/s41598-026-36062-8 (PMC12891725; doi:10.1038/s41598-026-36062-8)
Supplement: Supplementary file 2 — Supplementary Material 2 [file 41598_2026_36062_MOESM2_ESM.docx]

Table S2. Sensitivity analysis of the association between BRI and epilepsy after excluding participants taking valproate

|  | OR | 95%CI | P |
| --- | --- | --- | --- |
| Model 1 | 1.11 | (1.05,1.18) | <0.01 |
| Model 2 | 1.11 | (1.04,1.18) | <0.01 |
| Model 3 | 1.10 | (1.03,1.17) | <0.01 |
| Model 4 | 1.08 | (1.01,1.15) | 0.03 |

Participants reporting valproate were excluded (n=6). ORs and 95% CIs for epilepsy are shown per 1-unit increase in BRI. Model 1 is unadjusted; Model 2 is adjusted for age, sex, and race; Model 3 is additionally adjusted for education level, the ratio of family income to poverty, smoking status, and alcohol consumption; Model 4 is further adjusted for diabetes and hypertension. Abbreviations: BRI, body roundness index; OR, odds ratio; CI, confidence interval.
