# Supplementary material for: Elevated body roundness index and epilepsy prevalence: a cross-sectional study
Source: Sci Rep. 2026 Jan 19;16:5685. doi: 10.1038/s41598-026-36062-8 (PMC12891725; doi:10.1038/s41598-026-36062-8)
Supplement: Supplementary file 4 — Supplementary Material 4 [file 41598_2026_36062_MOESM4_ESM.docx]

Table S4. Sensitivity analysis of the association between BRI tertiles and epilepsy after excluding participants taking valproate

|  | BRI | OR | 95%CI | *P* |
| --- | --- | --- | --- | --- |
| Model 1 | Q1 | ref | ref | ref |
|  | Q2 | 1.70 | (1.05,2.79) | 0.03 |
|  | Q3 | 2.16 | (1.37,3.50) | 0.001 |
| Model 2 | Q1 | ref | ref | ref |
|  | Q2 | 1.70 | (1.04,2.84) | 0.04 |
|  | Q3 | 2.13 | (1.32,3.50) | 0.002 |
| Model 3 | Q1 | ref | ref | ref |
|  | Q2 | 1.70 | (1.03,2.84) | 0.04 |
|  | Q3 | 1.99 | (1.23,3.31) | 0.01 |
| Model 4 | Q1 | ref | ref | ref |
|  | Q2 | 1.60 | (0.97,2.68) | 0.07 |
|  | Q3 | 1.78 | (1.08,3.00) | 0.03 |

Participants reporting valproate were excluded (n=6). ORs and 95% CIs for epilepsy are shown for BRI tertiles, with Q1 [1.17–4.29] as the reference group, Q2 (4.29–6.22], and Q3 (6.22–23.48]. Model 1 is unadjusted; Model 2 is adjusted for age, sex, and race; Model 3 is additionally adjusted for education level, the ratio of family income to poverty, smoking status, and alcohol consumption; Model 4 is further adjusted for diabetes and hypertension. Abbreviations: BRI, body roundness index; OR, odds ratio; CI, confidence interval.
