# Supplementary material for: Elevated body roundness index and epilepsy prevalence: a cross-sectional study
Source: Sci Rep. 2026 Jan 19;16:5685. doi: 10.1038/s41598-026-36062-8 (PMC12891725; doi:10.1038/s41598-026-36062-8)
Supplement: Supplementary file 5 — Supplementary Material 5 [file 41598_2026_36062_MOESM5_ESM.docx]

Table S5. Sensitivity analysis of the association between BRI tertiles and epilepsy after excluding participants taking carbamazepine

|  | BRI | OR | 95%CI | *P* |
| --- | --- | --- | --- | --- |
| Model 1 | Q1 | ref | ref | ref |
|  | Q2 | 1.66 | (0.99,2.82) | 0.06 |
|  | Q3 | 2.27 | (1.41,3.78) | 0.001 |
| Model 2 | Q1 | ref | ref | ref |
|  | Q2 | 1.63 | (0.97,2.81) | 0.07 |
|  | Q3 | 2.15 | (1.31,3.64) | 0.003 |
| Model 3 | Q1 | ref | ref | ref |
|  | Q2 | 1.62 | (0.96,2.80) | 0.08 |
|  | Q3 | 1.98 | (1.19,3.38) | 0.01 |
| Model 4 | Q1 | ref | ref | ref |
|  | Q2 | 1.54 | (0.90,2.67) | 0.12 |
|  | Q3 | 1.82 | (1.08,3.16) | 0.03 |

Participants reporting carbamazepine were excluded (n=23). ORs and 95% CIs for epilepsy are shown for BRI tertiles, with Q1 [1.17–4.29] as the reference group, Q2 (4.29–6.22], and Q3 (6.22–23.48]. Model 1 is unadjusted; Model 2 is adjusted for age, sex, and race; Model 3 is additionally adjusted for education level, the ratio of family income to poverty, smoking status, and alcohol consumption; Model 4 is further adjusted for diabetes and hypertension. Abbreviations: BRI, body roundness index; OR, odds ratio; CI, confidence interval.
